# The randomized ZIPANGU trial of ranibizumab and adjunct laser for macular edema following branch retinal vein occlusion in treatment-naïve patients

**DOI:** 10.1038/s41598-020-79051-1

**Published:** 2021-01-12

**Authors:** Toshinori Murata, Mineo Kondo, Makoto Inoue, Shintaro Nakao, Rie Osaka, Chieko Shiragami, Kenji Sogawa, Akikazu Mochizuki, Rumiko Shiraga, Yohei Ohashi, Takeumi Kaneko, Chikatapu Chandrasekhar, Akitaka Tsujikawa, Motohiro Kamei

**Affiliations:** 1 Department of Ophthalmology, Shinshu University School of Medicine, 3-1-1 Asahi, Matsumoto, Nagano, Japan; 2 Department of Ophthalmology, Mie University Graduate School of Medicine, Mie, Japan; 3 Department of Ophthalmology, Kyorin Eye Center, Kyorin University School of Medicine, Tokyo, Japan; 4 Department of Ophthalmology, Graduate School of Medical Sciences, Kyushu University, Fukuoka, Japan; 5 Department of Ophthalmology, Kagawa University Faculty of Medicine, Kagawa, Japan; 6 Department of Ophthalmology, Asahikawa Medical University, Hokkaido, Japan; 7 Novartis Pharma K.K., Tokyo, Japan; 8 Novartis Healthcare Pvt. Ltd., Hyderabad, India; 9 Department of Ophthalmology and Visual Sciences, Kyoto University Graduate School of Medicine, Kyoto, Japan; 10 Department of Ophthalmology, Aichi Medical University, Aichi, Japan

**Keywords:** Macular degeneration, Retinal diseases

## Abstract

The ZIPANGU study assessed the efficacy and safety of ranibizumab as a one loading dose + *pro re nata* (one + PRN) regimen with/without focal/grid laser among treatment-naïve patients suffering from macular edema (ME) following branch retinal vein occlusion (BRVO). ZIPANGU was a phase IV, prospective, randomized, open-label, active-controlled, 12-month, two-arm, multicenter study. Treatment-naïve patients with visual impairment (19–73 letters) caused by ME, defined as central subfield thickness (CSFT) > 300 µm, due to BRVO were randomly assigned to ranibizumab monotherapy (n = 29) or combination therapy (ranibizumab + focal/grid short-pulse laser, n = 30). The primary endpoint was the number of ranibizumab injections. Secondary endpoints were mean changes in best-corrected visual acuity (BCVA) and CSFT, and safety. There were no statistically significant differences in the mean number of ranibizumab injections between monotherapy (4.3 injections) vs. combination (4.1 injections) therapy, or in CSFT. BCVA improvement in the monotherapy arm (22.0 letters) was better than the combination therapy arm (15.0 letters) (p = 0.035). Overall, both regimens appeared to be safe and well tolerated. One + PRN ranibizumab is safe and efficacious in treatment-naïve patients with ME secondary to BRVO. A conjunctive laser treatment did not lead to better functional outcomes or fewer ranibizumab injections.

## Introduction

Retinal vein occlusion (RVO), which includes branch retinal vein occlusion (BRVO), central retinal vein occlusion (CRVO), or hemi-CRVO, is the second most common vision-threatening vascular disorder of the retina after diabetic retinopathy^1^. Major risk factors for BRVO include hypertension, arteriosclerosis, and diabetes^2,3^. Asian, including Japanese, and Hispanic individuals have been reported to have a higher prevalence of these risk factors (e.g., hypertension), which predisposes them to have an increased risk of BRVO, compared with Western populations^3,4^. Interestingly, according to data from the HISAYAMA study, a prospective large-scale cohort study in Japan, BRVO has higher prevalence (2.0%)^5^ than diabetic retinopathy (1.9%)^6^ and exudative age-related macular degeneration (0.87%)^7^.

Macular edema (ME) is a major complication that causes central vision loss in patients with BRVO^1,8–10^. ME arises from the breakdown of the blood–retinal barrier, which is mediated, at least in part, by vascular endothelial growth factor (VEGF)^11–13^. Several anti-VEGF agents are now available for ocular use, and data from clinical studies^14–19^ and meta-analyses^20,21^ support their first-line use for the treatment of BRVO.

The anti-VEGF agent ranibizumab (LUCENTIS) is approved for the treatment of ME due to RVO in more than 100 countries worldwide, including Japan, as an intravitreal injection of 0.5 mg. However, patients receiving anti-VEGF therapy may have repeated recurrences of ME, and some patients are poor responders to this type of treatment^22^. For example, in the BRIGHTER study, more than 40% of patients in the ranibizumab monotherapy arm required > 12 injections over 24 months^23^. It has also been reported that frequent and prolonged exposure to anti-VEGF agents may lead to systemic adverse events (AEs)^24,25^. Repeated treatment also places a high co-pay financial burden on BRVO patients. In addition to increasing treatment efficacy, there is a need for further treatments that minimize the risk of systemic adverse events and the patient’s financial burden by reducing the number of intravitreal injections of anti-VEGF agents.

Before anti-VEGF agents were approved, focal/grid laser was the standard treatment for ME secondary to BRVO^1,22^. While some clinical studies demonstrated that macular focal/grid laser combined with an anti-VEGF agent may have the potential to reduce the number of anti-VEGF injections and maintain^26^ or improve treatment efficacy^27–29^, two multicenter randomized controlled clinical trials with large cohorts (the RELATE study^30^ and BRIGHTER study^23^) concluded that the use of additional laser did not have add-on efficacy for visual acuity (VA), edema resolution, or number of ranibizumab injections.

The ZIPANGU study was initiated to assess the efficacy and safety of ranibizumab as a one loading dose + *pro re nata* (one + PRN) regimen with/without focal/grid laser among treatment-naïve patients suffering from ME following BRVO. Although prior studies have explored similar outcomes, the ZIPANGU study aimed to clarify the optimal ranibizumab regimen via clearly stipulated methodologic modifications. The RELATE study investigated the effect of adjunct scatter laser with grid laser^30^. In the BRIGHTER study, adjunct laser was allowed at the doctors’ discretion; this was presumably focal/grid laser only, without scatter laser, but the technique was not defined clearly in the protocol^23^. Consequently, in the ZIPANGU study, to specifically investigate the effect of focal/grid laser on the number of ranibizumab injections, the method for delivery of additional laser was clearly defined in the protocol (focal/grid laser to capillary nonperfusion within the vascular arcades or residual ME detected by a macular thickness on optical coherence tomography [OCT]). To ascertain the effects of focal/grid laser, concomitant scatter laser was prohibited during the 1-year study period.

Moreover, in most previous large, multicenter, randomized clinical trials investigating the efficacy of ranibizumab, three to six monthly injections were administered during the induction phase. The number of monthly injections administered in the induction phase was three in BRIGHTER^23^ and six in BRAVO^31^ and RELATE^30^ studies. However, Miwa et al.^32^ reported that one + PRN and three + PRN regimens achieved similar 12-month functional outcomes. They concluded that the one + PRN regimen could reduce patients’ physical and economic burdens to a great extent by reducing the overall number of ranibizumab injections without hampering the vision-improving effect of ranibizumab^32^. Similarly, a retrospective, single-center exploration of one + PRN and three + PRN regimens reported no significant differences in anatomical or functional results at 12 months^33^.

The main objective of the present study was to examine whether two specific modifications of the regimen (adjunctive focal/grid laser and a one + PRN regimen of ranibizumab injections during the induction phase) in treatment-naïve Japanese patients could reduce the total number of ranibizumab injections compared with previous clinical studies of ranibizumab monotherapy with three to six injections during the induction phase. The efficacy, in terms of improving vision, and the safety of the regimen were also investigated.

## Results

### Patients

**Figure 1:**
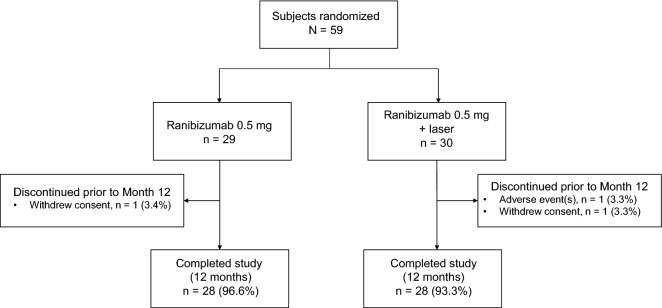
Patient disposition (randomized set).

In total, 59 patients were randomly assigned to treatment, 29 to ranibizumab monotherapy and 30 to ranibizumab plus laser combination therapy (Fig. 1). More than 90% of patients in each arm completed the study. One patient in each arm withdrew consent prior to study completion, and one patient in the combination treatment arm discontinued due to a serious AE (left putamen bleeding).

Patient baseline demographics are reported in Table 1. All patients were Japanese. The mean age was 66.8 years, and more patients were aged ≥ 65 years (61.0%) than < 65 years (39.0%). Baseline characteristics were generally well-balanced between treatment arms, except for sex: more female patients were randomly assigned to treatment with combination therapy (63.3%), and more male patients received monotherapy (65.5%).

**Table 1:** Patient baseline demographics and ocular characteristics (randomized set).

Variable	Ranibizumab 0.5 mg n = 29	Ranibizumab 0.5 mg + laser n = 30	Total N = 59
**Age, years**			
Mean (SD)	66.8 (9.9)	66.7 (9.8)	66.8 (9.8)
< 65 years, n (%)	12 (41.4)	11 (36.7)	23 (39.0)
≥ 65 years, n (%)	17 (58.6)	19 (63.3)	36 (61.0)
**Sex, n (%)**			
Male	19 (65.5)	11 (36.7)	30 (50.8)
Female	10 (34.5)	19 (63.3)	29 (49.2)
Visual acuity, letters, mean (SD)	52.9 (13.4)	54.0 (9.8)	53.5 (11.6)
**Visual acuity stratification group, letters, n (%)**			
≤ 39	4 (13.8)	4 (13.3)	8 (13.6)
40–59	15 (51.7)	15 (50.0)	30 (50.8)
≥ 60	10 (34.5)	11 (36.7)	21 (35.6)
Central subfield thickness, μm, mean (SD)	563.3 (146.9)	553.3 (182.4)	558.2 (164.6)
**Type of perfusion, n (%)**			
Ischemic	17 (58.6)	19 (63.3)	36 (61.0)
Non-ischemic	12 (41.4)	10 (33.3)	22 (37.3)
Missing	0 (0.0)	1 (3.3)	1 (1.7)
Presence of macular ischemia, n (%)	16 (55.2)	17 (56.7)	33 (55.9)
Presence of ≥ 10 Da retinal non-perfusion area	10 (34.5)	13 (43.3)	23 (39.0)
**Time since first BRVO symptoms, months**			
Mean (SD)	2.0 (1.0)	2.4 (1.3)	2.2 (1.2)
< 3 months, n (%)	23 (79.3)	21 (70.0)	44 (74.6)
≥ 3 months to < 6 months, n (%)	6 (20.7)	9 (30.0)	15 (25.4)
Presence of sub-retinal fluid, n (%)	17 (58.6)	24 (80.0)	41 (69.5)
Macular BRVO, n (%)	0 (0)	0 (0)	0 (0)

*BRVO* branch retinal vein occlusion, *SD* standard deviation.

Baseline ocular characteristics are also reported in Table 1. Mean VA was 53.5 letters, 13.6% of patients had mean VA ≤ 39 letters, and mean central subfield thickness was 558.2 μm. More patients had ischemic perfusion (61.0%) vs. non-ischemic perfusion (37.3%). The mean time since onset of BRVO symptoms was 2.2 months; no patients had symptoms for ≥ 6 months.

### Primary study outcome

**Figure 2:**
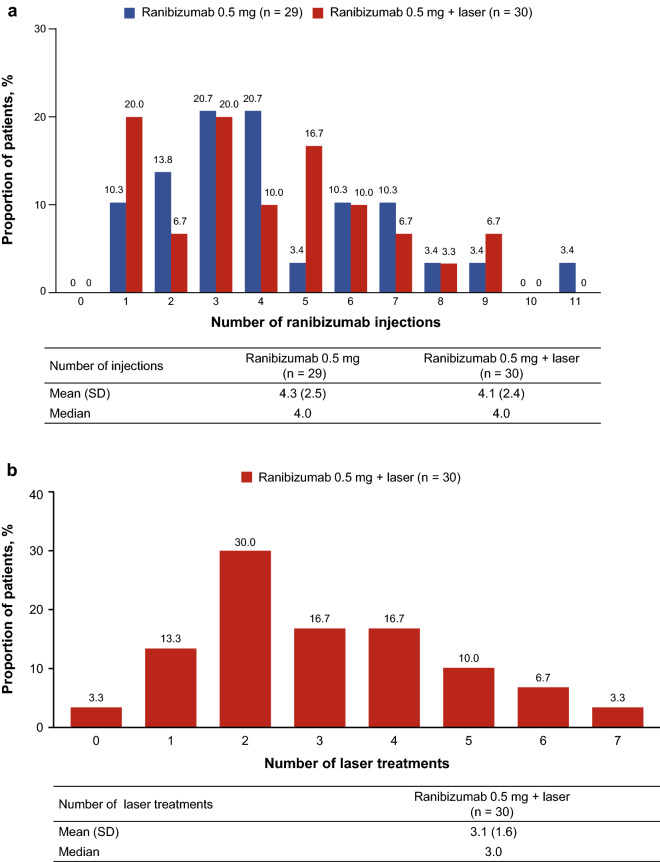
Treatments (full analysis set, observed). (**a**) Number of ranibizumab treatments from Day 1 to Month 11. (**b**) Number of laser treatments. *SD* standard deviation.

The primary study endpoint was to examine whether ranibizumab plus focal/grid laser reduces the mean number of ranibizumab injections compared with ranibizumab monotherapy. There was no statistically significant difference (p = 0.37) between the mean (standard deviation [SD]) number of injections administered over 12 months in the two treatment arms: monotherapy 4.3 (2.5) vs. combination therapy 4.1 (2.4); difference − 0.2 (0.6), 95% confidence interval (CI): − 1.5, 1.1 (Fig. 2a). The mean (SD) number of laser treatments was 3.1 (1.6) (Fig. 2b).

### Secondary outcomes: efficacy

**Figure 3:**
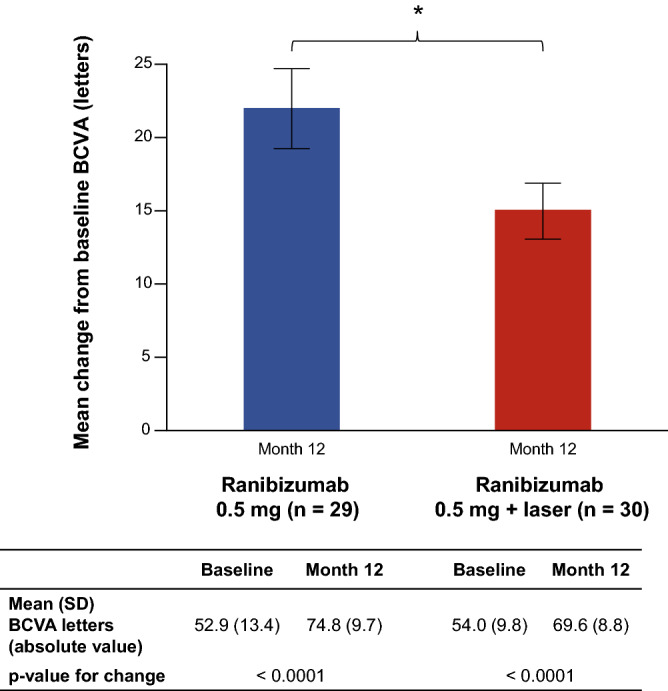
Mean change from baseline in BCVA letter score in the study eye at Month 12 (full analysis set, LOCF). *BCVA* best corrected visual acuity, *LOCF* last observation carried forward, *SD* standard deviation. *p < 0.05.

Each type of treatment, as assessed by best corrected visual acuity (BCVA), showed significant improvement from baseline at Month 12. The mean (SD) Early Treatment in Diabetic Retinopathy Study (ETDRS) letters was 74.8 (9.7) for monotherapy (mean change from baseline 22.0 [14.7], p < 0.0001) and 69.6 (8.8) for combination therapy (mean change from baseline 15.0 [10.3], p < 0.0001); p-value for treatment difference between monotherapy and combination at Month 12 = 0.035 (Fig. 3). The mean (SD) log of the minimal angle of resolution (logMAR) was 0.1 (0.2) for monotherapy (mean change from baseline − 0.5 [0.3]) vs. 0.1 (0.2) for combination therapy (mean change from baseline − 0.4 [0.2]); p-value for monotherapy vs. combination = 0.271 (see Supplementary Table S1 online).

Details of BCVA improvements according to attainment of ETDRS letters at Month 12 are reported in Supplementary Fig. S1 online. At Month 12, significantly more patients (p < 0.05) achieved a BCVA score of ≥ 85 letters and an improvement of ≥ 30 letters in the monotherapy arm than in the combination therapy arm, while 13 patients in the monotherapy arm and eight patients in the combination therapy arm achieved a BCVA score of ≤ 0.0 logMAR (data not shown). In a subgroup analysis by baseline BCVA (letters), the change from baseline at Month 12, in patients with baseline BCVA < 60 letters, was significantly better with monotherapy compared with combination therapy (p < 0.05) (see Supplementary Fig. S2 online).

**Figure 4:**
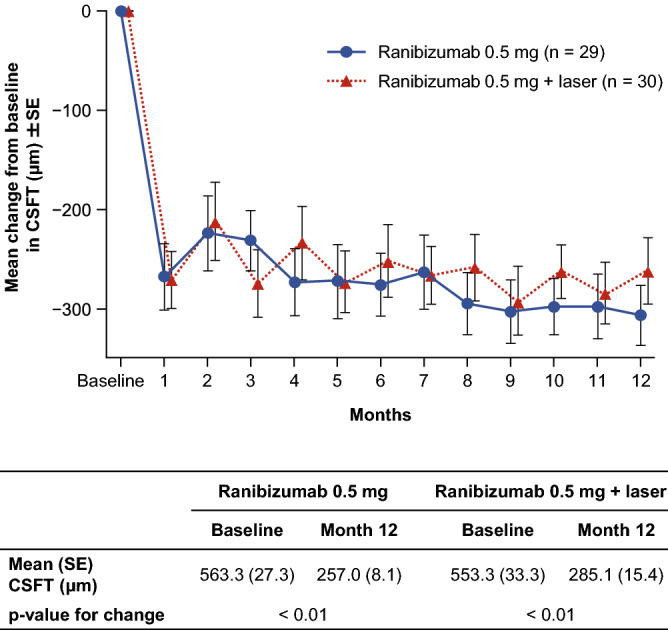
Mean change from baseline in CSFT (µm) ± SE (full analysis set, LOCF). *CSFT* central subfield thickness, *LOCF* last observation carried forward, *SE* standard error.

There were no significant differences between the two treatment arms in central subfield thickness (CSFT) at Month 12 (Fig. 4). The mean (standard error) change from baseline at Month 12 was − 306.3 (30.5) for monotherapy vs. − 261.3 (33.5) for combination therapy.

### Exploratory efficacy analyses

The retinal sensitivity data are shown in Supplementary Table S2 online. In both monotherapy and combination therapy arms, retinal sensitivity improved at Month 12 compared to the baseline. There was no significant sensitivity loss even in the affected side of the macula where short-pulse focal/grid laser was delivered.

Longitudinal changes in the number of foveal and macular leaking microaneurysms detected with fluorescein angiography within the 6 mm perifoveal subfield are shown in Supplementary Fig. S3 online. In the monotherapy arm, the number of microaneurysms increased with time. Conversely, in the combination arm, there was a tendency towards an increase from baseline in the number of microaneurysms at Month 6; subsequently, the numbers decreased by Month 12.

Linear regression of BCVA absolute value at Month 12 indicated that the presence of sub-retinal fluid at baseline was associated with visual outcomes in the monotherapy arm (p < 0.05).

### Secondary outcomes: safety

AEs (ocular and non-ocular) occurring in ≥ 2% of patients are reported in Table 2; there were no notable differences between treatment arms. Ocular AEs consisted largely of ocular surface changes, probably caused by the contact lens used during the laser treatment. Consequently, more patients in the combination arm reported ocular AEs (23.3%; 7/30 eyes) compared with the monotherapy arm (6.9%; 2/29 eyes). The only ocular AE that occurred in > 1 patient in either arm was ‘intraocular pressure increased’ in three patients (10.0%) in the combination arm. Three patients reported non-ocular serious AEs: one in the monotherapy arm (severe hematuria) and two in the combination arm (mild Bowen’s disease and severe left putamen bleeding); as previously noted, the left putamen bleeding event resulted in patient discontinuation. There were no serious ocular AEs or deaths reported in the study.

**Table 2:** Summary of adverse events (safety set).

Preferred term	Ranibizumab 0.5 mg n = 29	Ranibizumab 0.5 mg + laser n = 30
**Ocular AEs, n (%)**	2 (6.9)	7 (23.3)
Dry eye	1 (3.4)	1 (3.3)
Keratitis	1 (3.4)	0
Conjunctival hemorrhage	0	1 (3.3)
Corneal erosion	0	1 (3.3)
Intraocular pressure increased	0	3 (10.0)
Macular fibrosis	0	1 (3.3)
Seasonal allergy	0	1 (3.3)
**Non-ocular AEs, n (%)**	15 (51.7)	11 (36.7)
Nasopharyngitis	6 (20.7)	5 (16.7)
Headache	3 (10.3)	0
Hypertension	2 (6.9)	1 (3.3)
Nausea	2 (6.9)	1 (3.3)
Arthritis	1 (3.4)	0
Dizziness	1 (3.4)	1 (3.3)
Dry skin	1 (3.4)	0
Foot fracture	1 (3.4)	0
Hematuria	1 (3.4)	0
Hepatic function abnormal	1 (3.4)	0
Influenza	1 (3.4)	1 (3.3)
Periodontitis	1 (3.4)	0
Seasonal allergy	1 (3.4)	0
Tooth fracture	1 (3.4)	0
Urinary tract infection	1 (3.4)	0
Atrophic vulvovaginitis	0	1 (3.3)
Back pain	0	1 (3.3)
Bowen’s disease	0	1 (3.3)
Dysarthria	0	1 (3.3)
Erythema	0	1 (3.3)
Feeling abnormal	0	1 (3.3)
Hemiplegia	0	1 (3.3)
Migraine	0	1 (3.3)
Muscular weakness	0	1 (3.3)
Putamen hemorrhage	0	1 (3.3)
Tinnitus	0	1 (3.3)

*AE* adverse event.

## Discussion

Anti-VEGF therapy is currently the standard first-line treatment for ME secondary to BRVO^16–19,23,34,35^. However, frequent long-term exposure to anti-VEGF agents may lead to systemic AEs and places an economic burden on patients^24,25^. To improve the feasibility of this treatment, we investigated whether two specific modifications of the ranibizumab regimen could reduce patients’ physical and economic burden by decreasing the overall number of injections (primary endpoint). In addition, we examined whether a reduction in the number of ranibizumab injections, through specified treatment modifications, could potentially have a negative impact on the vision-improving effect of this drug, by assessing the secondary efficacy and safety endpoints.

In this study, we found that the first modification (the use of a conjunctive focal/grid laser) did not lead to fewer ranibizumab injections. There were no statistically significant differences in the overall number of ranibizumab injections between the ranibizumab monotherapy arm (4.3 ± 2.5 injections) and combination therapy arm of ranibizumab and focal/grid laser (4.1 ± 2.4 injections). Both monotherapy and combination therapy appeared to be safe and well tolerated in this patient population, and no new safety concerns were identified.

Although additional focal/grid laser did not contribute to the reduction of ranibizumab injections vs. ranibizumab monotherapy, the overall number of ranibizumab injections in both arms of this study did appear to be reduced compared with previous clinical trials that used ranibizumab. While direct comparisons are not possible, owing to data being analyzed at different time points and in heterogeneous settings, the mean number of injections in the ranibizumab monotherapy arm (one + PRN regimen) of the current ZIPANGU study (4.3 injections in 12 months) was lower compared with the number of injections reported in previous multicenter clinical trials: 8.4 injections in 12 months (six + PRN regimen) in the BRAVO study^14^ and 4.8 injections in 6 months (three + PRN regimen) in the BRIGHTER study^34^. It appears that the one + PRN regimen administered in the present study, at least in part, contributed to reducing the overall number of ranibizumab injections.

When aiming to reduce the number of anti-VEGF injections, it is also critical to maintain the vision improvements seen in the previous studies using anti-VEGF monotherapy^14,18,19,23,34,35^. In this study, a greater BCVA improvement was shown in the monotherapy arm (22.0 letters) than the combination therapy arm (15.0 letters). Of note, however, even the vision improvement in the combination arm (15.0 letters) of the present study was comparable to that reported in previous multicenter clinical trials of anti-VEGF monotherapy such as the BRIGHTER (15.5 letters with 11.4 ranibizumab injections in the three + PRN regimen^23^, VIBRANT (17.1 letters with 9 aflibercept injections in the six + bimonthly regimen^17^, and BRAVO (18.3 letters with 8.4 ranibizumab injections in the six + PRN regimen)^14^ studies.

When focusing on the ranibizumab monotherapy arms, it is of particular interest to note that the ZIPANGU monotherapy arm showed a comparable or even greater improvement in vision (22 letters) compared with the BRAVO (18.3 letters)^14^ and BRIGHTER (15.5 letters)^23^ studies, despite the fact that patients in the monotherapy arm of the ZIPANGU study (4.3 injections) received a lower number of ranibizumab injections than those in the BRAVO (8.4 injections) and BRIGHTER studies (11.4 injections). However, as mentioned previously, these comparisons must be interpreted with caution, due to the heterogeneous patient populations and differences in study designs and treatment regimens.

There are two possible reasons why patients in the ZIPANGU study may have obtained better vision gain compared with patients in the previous studies. First, only treatment-naïve patients were enrolled in the ZIPANGU study, while previous clinical trials with large cohorts that investigated the effect of anti-VEGF agents for ME secondary to BRVO, such as the BRAVO^14^, HORIZON^15^, VIBRANT^17^, RETAIN^18^, BLOSSOM^19^, and BRIGHTER^23^ studies, enrolled patients with previous treatment history, including prior anti-VEGF agents. Second, patients in the ZIPANGU study had a much shorter BRVO disease duration than those in previous studies, as described below. Generally, treatment-naïve patients have a shorter disease duration before the initial ranibizumab injection than those with a treatment history. A short disease duration provides favorable visual outcomes by allowing patients to be treated before they present permanent photoreceptor damage caused by persistent ME.

In the BLOSSOM study, which evaluated patients with ME secondary to BRVO during a 6-month follow-up, the effect of early ranibizumab injection was investigated by comparing a ranibizumab treatment arm (1.6-month disease duration before the initial ranibizumab injection) and a sham treatment arm (1.8-month disease duration before the initial sham injection)^19^. This study confirmed the importance of early treatment in achieving optimal visual outcomes in BRVO. However, in most previous randomized clinical trials that evaluated the efficacy of ranibizumab monotherapy as well as combination therapy with adjunctive laser treatment, the average disease duration before treatment initiation was much longer than that of the current ZIPANGU study (ZIPANGU: monotherapy 2.0, combination 2.4 months; BRIGHTER: monotherapy 10.3, combination 9.2 months^34^; RABAMES: grid laser 5 months, ranibizumab monotherapy 5.1 months, combination 6.0 months^36^; and RELATE: 12.7 months^30^). Consequently, we consider that the short disease duration before the initial ranibizumab injection in treatment-naïve patients of the ZIPANGU study may have led to better vision improvement and reduction in the number of ranibizumab injections. These outcomes have ramifications for real-world clinical practice, suggesting that earlier treatment may be a key criterion for improving visual acuity outcomes with a reduced number of ranibizumab injections using a one + PRN regimen.

In this study, BCVA improvement in the monotherapy arm (22.0 letters) was better than that of the combination therapy arm (15.0 letters) (p = 0.035). These data suggest that the use of focal/grid laser, instead of acting synergistically to improve outcomes as we originally thought, actually suppressed the BCVA improvement induced by ranibizumab injections. This potential adverse effect of adjunct focal/grid laser on vision improvement is in contrast to the data from the BRIGHTER study conducted in European countries, Australia, and Canada^23^. In the BRIGHTER study, the authors concluded that the addition of laser (17.3 letters) to ranibizumab monotherapy (15.5 letters) had no impact on BCVA changes over 24 months^23^. We consider that this difference can be attributed to the laser methodology used. In the BRIGHTER study, adjunct laser was allowed at the doctors’ discretion, but the technique was not clearly defined in the protocol^23^. The mean (SD) number of laser treatment was 1.0 (0.57) in 24 months, and 14.2% of the patients in the ranibizumab + laser group never underwent adjunct laser treatment^23,34^. Conversely, the criteria for laser administration in the ZIPANGU study were clearly defined. All patients in the ranibizumab + laser arm who completed 12 months of follow up underwent at least one session of adjunct laser, and the mean (SD) number of adjunct laser treatments in ZIPANGU was 3.1 (1.6). These data suggest that the use of adjunct laser in some patients with macular edema secondary to BRVO actually resulted in overtreatment, which in turn led to smaller improvements in vision compared with ranibizumab monotherapy.

The ZIPANGU study has several limitations which must be considered. The relatively small number of patients and the open-label design of the ZIPANGU study are the main limitations, although treatment masking for the vision examiner evaluating BCVA was employed to reduce the level of bias as much as possible in the secondary efficacy outcomes. Moreover, as described above, we acknowledge that focal/grid laser application in our study did not meet the ETDRS guidelines, which form the basis of US and European standard treatments.

In conclusion, the results of the ZIPANGU study did not demonstrate the clinical utility of adding focal/grid laser treatment to ranibizumab monotherapy. These results support and confirm the use of one + PRN ranibizumab monotherapy in patients with ME secondary to BRVO at least in the first year of treatment, and suggest that clinicians need to carefully consider appropriate patient selection and timing before choosing to administer focal/grid laser treatment. One + PRN ranibizumab injection appeared to result in comparable or even better vision recovery than standard monthly injection regimens, provided the patients are treatment-naïve and start ranibizumab injections shortly after the onset of BRVO.

## Methods

### Study design

**Figure 5:**
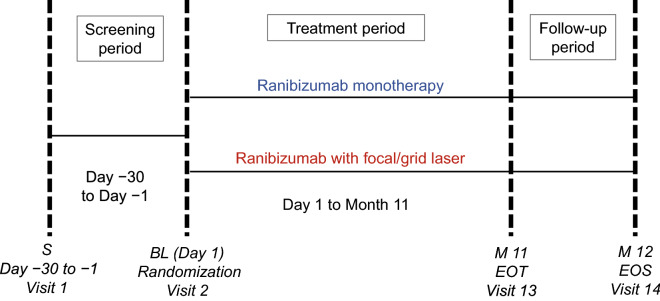
Design of the phase IV, randomized, open-label, active-controlled, 2-arm, multicenter ZIPANGU study. *BL* baseline, *EOT* end of treatment, *EOS* end of the study, *M* month, *S* screening.

The ZIPANGU study (NCT02953938, date of registration 03/11/2016) was a phase IV, randomized, open-label, active-controlled, 12-month, two-arm, multicenter clinical study (Fig. 5). The study started on 15 December 2016 (date on which the first patient was enrolled) and completed on 28 December 2018 (date of the last visit of the last patient). Patients visited the study centers at screening and baseline (Day 1), and monthly thereafter (Month 1 to Month 12). Although investigators and patients were aware of the treatment, the vision examiner who assessed BCVA was blinded to treatment.

The study was conducted in compliance with the 1964 Declaration of Helsinki and its later amendments, “Ethical Guidelines on Medical Research for Humans” (Japan Ministry of Health, Labour and Welfare), and in accordance with the International Conference on Harmonization E6 guideline for Good Clinical Practice. Approval for the study protocol and all associated documentation was obtained from the Institutional Review Board/Independent Ethics Committee at the following study centers: Shinshu University School of Medicine, Mie University Graduate School of Medicine, Kyorin University School of Medicine, Kyushu University Graduate School of Medical Sciences, Kagawa University Faculty of Medicine, Asahikawa Medical University, and Aichi Medical University. Patients provided written informed consent prior to inclusion in the study.

### Treatment

Patients were randomly assigned by interactive response technology, in a 1:1 ratio, to one of the two treatment arms: Arm 1, receiving ranibizumab monotherapy, and Arm 2, receiving ranibizumab plus focal/grid short-pulse laser combination therapy. The focal/grid method used here is a modified version of the ETDRS procedure and incorporates a short-pulse laser to minimize retinal tissue damage, thus avoiding atrophic creep. Randomization was balanced by VA (< 0.3 or ≥ 0.3, assessed by decimal VA/58 letters by ETDRS chart^37^).

Ranibizumab (0.5 mg) was administered as a one + PRN regimen. This was defined as an intravitreal injection on Day 1, with further treatments as needed (according to the prespecified criteria, with a minimum gap of 30 ± 7 days between injections). Retreatment criteria were applied monthly if one or more of the following items were met after Day 1: CSFT ≥ 300 μm; CSFT increase of ≥ 20% compared with the minimum value during the treatment period; and loss of VA due to disease activity secondary to BRVO, based on the investigator’s judgement.

Laser treatment was administered to the target within vascular arcades based on the ETDRS guidelines^38,39^. Details are provided in the Supplementary materials. Focal/grid laser was required to be performed at least 30 min before the ranibizumab injection or could be deferred for up to 14 days after the injection. Based on the investigator’s judgement, initial laser treatment could be postponed until dense macular hemorrhage and/or severe retinal edema (thickness > 400 μm on the OCT image) were resolved. Following the complete application of the initial laser treatment, if an area with thickness > 350 μm appeared on the OCT image, further laser treatment was administered at minimal intervals of 30 ± 7 days.

### Patients

The inclusion criteria (measured at screening and confirmed at baseline [Day 1]) were male or female patients aged ≥ 20 years, diagnosis of visual impairment caused by ME secondary to BRVO, BCVA score at screening and baseline between 0.5 and 0.05 decimal with Landolt C chart (73–19 letters by ETDRS testing) and logMAR score of 0.3–1.3^37^, increased CSFT (> 300 μm on Day 1), and duration of vision deterioration ≤ 6 months at screening. Key exclusion criteria were patients with a history of stroke or myocardial infarction within 3 months before screening, who used any anti-angiogenic drug on a contralateral eye within 3 months before baseline or on a treatment eye before baseline, and who had panretinal laser photocoagulation therapy within 1 month before baseline or focal/grid laser therapy before baseline. Further inclusion criteria applicable to cases when both eyes were eligible, and additional exclusion criteria are provided in the Supplementary materials.

### Study outcomes

The primary study objective was to evaluate whether a one + PRN regimen of ranibizumab combined with focal/grid short-pulse laser reduces the burden of frequent ranibizumab injections compared with ranibizumab monotherapy. The primary endpoint was the difference in the mean number of ranibizumab injections administered up to Month 11 between the two treatment arms.

Secondary study objectives were to evaluate the efficacy of each arm, assessed by mean change in BCVA and CSFT (secondary efficacy endpoints) from baseline (Day 1) to Month 12, and to evaluate the safety of each arm, assessed by the type, frequency, and severity of ocular and non-ocular AEs. BCVA of the study eyes was assessed at every visit using decimal VA testing charts at an initial testing distance of 5 m. BCVA was also assessed at baseline and at the Month 6 and Month 12 visits using ETDRS VA testing charts at an initial testing distance of 4 m. OCT images were obtained using spectral-domain equipment at every study visit and patients were assessed using the same machine throughout the course of the study; the CSFT represented the average retinal thickness of the circular area with 1 mm diameter around the foveal center (ETDRS central subfield). Safety was assessed by the type, frequency, and severity of AEs, plus an assessment of vital signs and laboratory test results. AEs were coded using terminology from the Medical dictionary for regulatory activities version 21.1.

Exploratory study objectives were to evaluate the mean change in retinal sensitivity using microperimetry from baseline to Month 12, and to examine the efficacy of each arm in relation to patient baseline characteristics assessed by the mean BCVA change from Month 1 through Month 12 (exploratory endpoints). Longitudinal changes in the number of foveal and macular leaking microaneurysms at Months 6 and 12 were also evaluated. Full details of all study measures can be found in the Supplementary materials.

### Statistical methods

To calculate the patient number required for this study, a difference between arms of two injections and an SD of 2.32 (based on the CAVNAV study^29^) was assumed. Based on the non-parametric Wilcoxon-Mann Whitney test for the difference in means, this would require 25 patients per arm with 80% power and a 0.025 significance level (one-sided). Assuming a discontinuation rate of ~ 20%, approximately 70 patients were screened to provide at least 56 eligible patients to commence study treatment. All sample size calculations were performed using EAST 6.0 (Cytel, Cambridge, MA, USA).

The full analysis set (FAS) included all randomized patients who received at least one ranibizumab injection; the safety set included all patients who received at least one ranibizumab injection and had at least one post-baseline safety assessment. Summary statistics are reported for continuous variables (N, mean, median, SD, and standard error), and the number and percentage of patients for categorical or binary variables.

The primary analysis was conducted at Month 12 within the FAS using observed data at an alpha level of 0.025. The statistical hypothesis testing of the number of ranibizumab treatments was based on a stratified Cochran–Mantel–Haenszel (CMH) test. Stratification was performed based on categories of baseline decimal VA (< 0.3 or ≥ 0.3). The difference in the mean number of injections, 95% CI of the difference, and one-sided p-value of the CMH test are reported. No imputation for missing data was required for the primary endpoint.

For the secondary efficacy analyses, the mean change in BCVA (letters) at baseline, Month 6, and Month 12 were compared based on an analysis of variance (ANOVA) model with treatment group, and stratification based on categories of baseline decimal VA (< 0.3 or ≥ 0.3). The least-squares (LS) mean, difference of LS means, their 95% CI, and the p-value (related to the null hypothesis that the difference in mean change is zero) were calculated. The mean change in BCVA (logMAR) from Month 1 through Month 12 was compared with baseline using a last observation carried forward (LOCF) approach. Endpoints related to the number and proportion of patients with BCVA letter gain or loss from baseline were analyzed via a stratified CMH test with stratification factors as described for the primary endpoint. The mean change in CSFT, as assessed by OCT imaging, from Month 1 through Month 12 compared with baseline was compared based on the ANOVA model, using the FAS and LOCF. P-values were 2-sided unless otherwise specified. All analyses were performed using SAS version 9.4 (SAS Institute Inc., Cary, NC, USA).

## Supplementary Information

Supplementary Information.
