# Author Correction: The randomized ZIPANGU trial of ranibizumab and adjunct laser for macular edema following branch retinal vein occlusion in treatment‑naïve patients

**DOI:** 10.1038/s41598-021-93187-8

**Published:** 2021-07-07

**Authors:** Toshinori Murata, Mineo Kondo, Makoto Inoue, Shintaro Nakao, Rie Osaka, Chieko Shiragami, Kenji Sogawa, Akikazu Mochizuki, Rumiko Shiraga, Yohei Ohashi, Takeumi Kaneko, Chikatapu Chandrasekhar, Akitaka Tsujikawa, Motohiro Kamei

**Affiliations:** 1 Department of Ophthalmology, Shinshu University School of Medicine, 3‑1‑1 Asahi, Matsumoto, Nagano, Japan; 2 Department of Ophthalmology, Mie University Graduate School of Medicine, Mie, Japan; 3 Department of Ophthalmology, Kyorin Eye Center, Kyorin University School of Medicine, Tokyo, Japan; 4 Department of Ophthalmology, Graduate School of Medical Sciences, Kyushu University, Fukuoka, Japan; 5 Department of Ophthalmology, Faculty of Medicine, Kagawa University, Kagawa, Japan; 6 Department of Ophthalmology, Asahikawa Medical University, Hokkaido, Japan; 7 Novartis Pharma K.K., Tokyo, Japan; 8 Novartis Healthcare Pvt. Ltd., Hyderabad, India; 9 Department of Ophthalmology and Visual Sciences, Kyoto University Graduate School of Medicine, Kyoto, Japan; 10 Department of Ophthalmology, Aichi Medical University, Aichi, Japan

Correction to: *Scientific Reports* 10.1038/s41598-020-79051-1, published online 12 January 2021

The original version of this Article contained errors in the Discussion section.

As a result,

“While direct comparisons are not possible, owing to data being analyzed at different time points and in heterogeneous settings, the mean number of injections in the ranibizumab monotherapy arm (one + PRN regimen) of the current ZIPANGU study (4.3 injections in 12 months) was lower compared with the number of injections reported in previous multicenter clinical trials: 8.5 injections in 12 months (six + PRN regimen) in the BRAVO study^35^ and 4.8 injections in 6 months (three + PRN regimen) in the BRIGHTER study^34^.”

now reads:

“While direct comparisons are not possible, owing to data being analyzed at different time points and in heterogeneous settings, the mean number of injections in the ranibizumab monotherapy arm (one + PRN regimen) of the current ZIPANGU study (4.3 injections in 12 months) was lower compared with the number of injections reported in previous multicenter clinical trials: 8.4 injections in 12 months (six + PRN regimen) in the BRAVO study^14^ and 4.8 injections in 6 months (three + PRN regimen) in the BRIGHTER study^34^.”

And,

“Of note, however, even the vision improvement in the combination arm (15.0 letters) of the present study was comparable to that reported in previous multicenter clinical trials of anti-VEGF monotherapy such as the BRIGHTER (15.5 letters with 14 ranibizumab injections in the three + PRN regimen^23^, VIBRANT (17.1 letters with 9 aflibercept injections in the six + bimonthly regimen^17^, and BRAVO (18.3 letters with 8.4 ranibizumab injections in the six + PRN regimen)^14^ studies.

When focusing on the ranibizumab monotherapy arms, it is of particular interest to note that the ZIPANGU monotherapy arm showed a comparable or even greater improvement in vision (22 letters) compared with the BRAVO (18.3 letters)^14^ and BRIGHTER (15.5 letters)^23^ studies, despite the fact that patients in the monotherapy arm of the ZIPANGU study (4.3 injections) received a lower number of ranibizumab injections than those in the BRAVO (8.4 injections) and BRIGHTER studies (14 injections).”

now reads:

“Of note, however, even the vision improvement in the combination arm (15.0 letters) of the present study was comparable to that reported in previous multicenter clinical trials of anti-VEGF monotherapy such as the BRIGHTER (15.5 letters with 11.4 ranibizumab injections in the three + PRN regimen^23^, VIBRANT (17.1 letters with 9 aflibercept injections in the six + bimonthly regimen^17^, and BRAVO (18.3 letters with 8.4 ranibizumab injections in the six + PRN regimen)^14^ studies.

When focusing on the ranibizumab monotherapy arms, it is of particular interest to note that the ZIPANGU monotherapy arm showed a comparable or even greater improvement in vision (22 letters) compared with the BRAVO (18.3 letters)^14^ and BRIGHTER (15.5 letters)^23^ studies, despite the fact that patients in the monotherapy arm of the ZIPANGU study (4.3 injections) received a lower number of ranibizumab injections than those in the BRAVO (8.4 injections) and BRIGHTER studies (11.4 injections).”

In addition, in the Supplementary Information file,

“Focal/grid short-pulse laser treatment was performed to suppress excess production of vascular endothelial growth factor (VEGF) by alleviating retinal ischemia. To achieve this, the entire capillary non-perfusion area in the vascular arcade was covered with focal/grid short-pulse laser treatment. OCT and fluorescein angiography were used to depict the capillary non-perfusion area; in cases where high-resolution OCT images could not be acquired due to the effects of retinal bleeding or edema, focal/grid short-pulse laser treatment of treatable lesions was performed in the area enclosed by the nasal border, the central fovea border, the peripheral border, and the temporal border.”

now reads:

“Grid short-pulse laser treatment was performed to suppress excess production of vascular endothelial growth factor (VEGF) by alleviating retinal ischemia. To achieve this, the entire capillary non-perfusion area in the vascular arcade was covered with grid short-pulse laser treatment. OCT and fluorescein angiography were used to depict the capillary non-perfusion area; in cases where high-resolution OCT images could not be acquired due to the effects of retinal bleeding or edema, grid short-pulse laser treatment of treatable lesions was performed in the area enclosed by the nasal border, the central fovea border, the peripheral border, and the temporal border.”

The original Supplementary Information file is provided below.

The original Article and its Supplementary Information file have been corrected.

## Supplementary Information

Supplementary Information.

