# Supplementary material for: The randomized ZIPANGU trial of ranibizumab and adjunct laser for macular edema following branch retinal vein occlusion in treatment-naïve patients
Source: Sci Rep. 2021 Jan 12;11:551. doi: 10.1038/s41598-020-79051-1 (PMC7804316; doi:10.1038/s41598-020-79051-1)
Supplement: Supplementary file 1 — Supplementary Information. [file 41598_2020_79051_MOESM1_ESM.pdf]

Supplementary Materials

The randomized ZIPANGU trial of ranibizumab and adjunct laser for macular edema following branch retinal vein occlusion in treatment-naïve patients

Toshinori Murata, Mineo Kondo, Makoto Inoue, Shintaro Nakao, Rie Osaka, Chieko Shiragami, Kenji Sogawa, Akikazu Mochizuki, Rumiko Shiraga, Yohei Ohashi, Takeumi Kaneko, Chikatapu Chandrasekhar, Akitaka Tsujikawa, Motohiro Kamei

Supplementary Methods

Laser treatment based on the Early Treatment Diabetic Retinopathy Study guidelines [1,2]

If there was little retinal bleeding and a treatable lesion was present, focal/grid short-pulse laser treatment was initially performed on Day 1. If the investigator determined that laser treatment could not be performed due to the observation of high-density retinal bleeding or severe retinal edema, laser treatment was postponed until the bleeding or edema was mitigated (retinal thickness < 400 μm with depiction as a yellow or green area on the optical coherence tomography [OCT] macular thickness map). Initial laser treatment was initiated as soon as possible depending on the mitigation pattern of the retinal bleeding or edema and could be divided into two or more sessions lasting into the following months until the capillary non-perfusion area in the vascular arcade was covered.

Grid short-pulse laser treatment was performed to suppress excess production of vascular endothelial growth factor (VEGF) by alleviating retinal ischemia. To achieve this, the entire capillary non-perfusion area in the vascular arcade was covered with grid short-pulse laser treatment. OCT and fluorescein angiography were used to depict the capillary non-perfusion area; in cases where high-resolution OCT images could not be acquired due to the

effects of retinal bleeding or edema, grid short-pulse laser treatment of treatable lesions was performed in the area enclosed by the nasal border, the central fovea border, the peripheral border, and the temporal border.

Direct short-pulse laser treatment was performed on individual microaneurysms, clusters of microaneurysms with fluid retention or leakage, and other leakage points such as enlarged capillaries. At the completion of direct short-pulse laser treatment, adequate coagulation of all treated microaneurysms was verified in case additional coagulation was deemed necessary (many microaneurysms first appear as a dark color but later take on a red color again).

Reference standards for laser photocoagulation conditions

	**Grid laser photocoagulation**	**Direct laser photocoagulation**
Wavelength	Green or yellow	Green or yellow
Spot size	50−100 μm	50 μm
Exposure time	0.02 or 0.03 s	0.02 or 0.03 s
Power/end point	The preferred appearance of grid laser is a slight whitening of the burns	The preferred appearance of direct laser is a whitening or darkening of the capillary abnormality and/or microaneurysm

Each parameter could be modified by the investigator. Subthreshold laser photocoagulation treatment was prohibited.

A contact lens with spot magnification of about one-power was to be used to keep the actual coagulation burn size on the retina similar to the setting of the photocoagulation machine. The contact lens was to provide a view that encompasses the entire vascular arcades to obtain precise distribution of the burns.

Additional details of inclusion criteria

If both eyes were eligible, the one with the worst visual acuity (VA) at screening was selected as the study eye, unless the other eye was deemed more appropriate by the investigator based on medical reasons. If the other eye presented with worsened VA due to macular edema secondary to retinal vein occlusion, it could also be treated with ranibizumab, laser treatment, or others, in line with Japanese regulations and at the discretion of the investigator; in this case, no anti-VEGF treatments other than ranibizumab were allowed, and bilateral treatment with ranibizumab could not be performed on the same day.

Study exclusion criteria

Study exclusion criteria were pregnancy, lactation, or inability or unwillingness to use appropriate contraception throughout the study and for 3 months after study treatment discontinuation; any medical or ocular condition which could put the safety of the patient at risk or confound the results of the study; history of malignancy (except localized basal cell skin carcinoma) within 5 years before screening; uncontrolled blood pressure; known hypersensitivity to ranibizumab or its excipients; inability to comply with study procedures; inability to obtain fundus photographs or fluorescein angiograms of sufficient quality to be analyzed; use of other investigational drugs within 30 days or 5 half-lives of baseline, whichever is longer; use of any systemic anti-VEGF drugs within 6 months before baseline; and current or planned use of systemic medications known to be toxic to the lens, retina, or optic nerve. Ocular conditions that would prevent study enrollment were as follows: active periocular or ocular infection or inflammation; uncontrolled glaucoma; cataract, aphakia, pseudoexfoliation, hemorrhage reducing VA, rhegmatogenous retinal detachment, macular hole, diabetic retinopathy, and diabetic maculopathy requiring treatment; choroidal neovascularization of any cause; brisk afferent pupillary defect; neovascularization of the iris or neovascular glaucoma; vitreomacular traction at screening or baseline; structural damage within 0.5 disc diameter of the center of the

macula; and history of herpetic ocular infection, ocular toxoplasmosis, or idiopathic central serous chorioretinopathy.

Ocular treatments that would prevent study enrollment were as follows: any intraocular procedure (including Yttrium-Aluminum-Garnet capsulotomy) within 2 months before baseline or anticipated within the next 12 months; topical ocular or systemic corticosteroids administered for at least 30 consecutive days within 6 months before screening; use of intra- or periocular corticosteroids within 3 months before screening; use of intraocular corticosteroid implants; history of optic neurotomy, sheathotomy, or filtration surgery at any time; and macular branch retinal vein occlusion.

Measures

Patient demographics and baseline characteristic data were collected, including age, sex, and retinal vein occlusion-specific baseline characteristics including date of branch retinal vein occlusion diagnosis. Efficacy assessments and pre-injection safety measures (tonometry, slit lamp, and fundus examinations) were required to be conducted before administration of ranibizumab with/without focal/grid laser.

Best-corrected visual acuity (BCVA) of the study eyes was assessed at every visit in a sitting position using decimal VA testing charts at an initial testing distance of 5 meters. BCVA was also assessed at baseline and at the Month 6 and Month 12 visits using Early Treatment in Diabetic Retinopathy Study (ETDRS) VA testing charts at an initial testing distance of 4 meters. For a BCVA subgroup analysis, the BCVA (letters) change from baseline at Month 12 was assessed in patients according to a baseline cut-off of < 60 letters; this value was based on the results of the BRIGHTER study [3].

OCT images were obtained using spectral-domain equipment at every study visit and patients were assessed using the same machine throughout the course of the study. Presence or absence of macular edema (ME) was evaluated by recording the thickness value of CSFT,

ETDRS inner or outer subfields, and the presence or absence of qualitative parameters (i.e., cysts, intraretinal fluid, subretinal fluid, and fibrosis).

The central subfield thickness (CSFT) represented the average retinal thickness of the circular area with 1 mm diameter around the foveal center (ETDRS central subfield). Color fundus photography and fundus autofluorescence images were obtained at screening, at Month 6, and at study completion, and were required to capture the presence or absence of ME, capillary leakage, and non-perfusion within the 6-mm perifoveal subfield.

OCT images were obtained using instruments such as AngioVue (Optovue Inc., Freemont, CA, USA); 6 × 6-mm areas, centered on the fovea, were scanned at every visit to capture the presence or absence of capillary dropout, microaneurysms, and collateral vessels of each superficial plexus and deep plexus within the 6-mm perifoveal subfield. Microperimetry was used to measure retinal sensitivity at baseline (Day 1), and at the end of the study.

Retinal sensitivity was measured at baseline and at Month 12 by fundus-monitored microperimetry, according to the standard practice of each investigator. Longitudinal changes in the number of foveal and macular leaking microaneurysms at Months 6 and 12 were evaluated using fluorescein angiography (FA), in conjunction with 7-field color fundus photography. Images were evaluated by the investigator according to their standard practice.

References

1. Early Treatment Diabetic Retinopathy Study Research Group. Photocoagulation for diabetic macular edema. Early Treatment Diabetic Retinopathy Study Report Number 1. *Arch. Ophthalmol.* **103**, 1796-1806 (1985).

2. Early Treatment Diabetic Retinopathy Study Research Group. Treatment techniques and clinical guidelines for photocoagulation of diabetic macular edema. Early Treatment Diabetic Retinopathy Study Report Number 2. *Ophthalmology.* **94**, 761-774 (1987).

3. Tadayoni, R. *et al*. Individualized stabilization criteria-driven ranibizumab versus laser in branch retinal vein occlusion: Six-month results of BRIGHTER. *Ophthalmology.* **123**, 1332-1344 (2016).

Supplementary figures

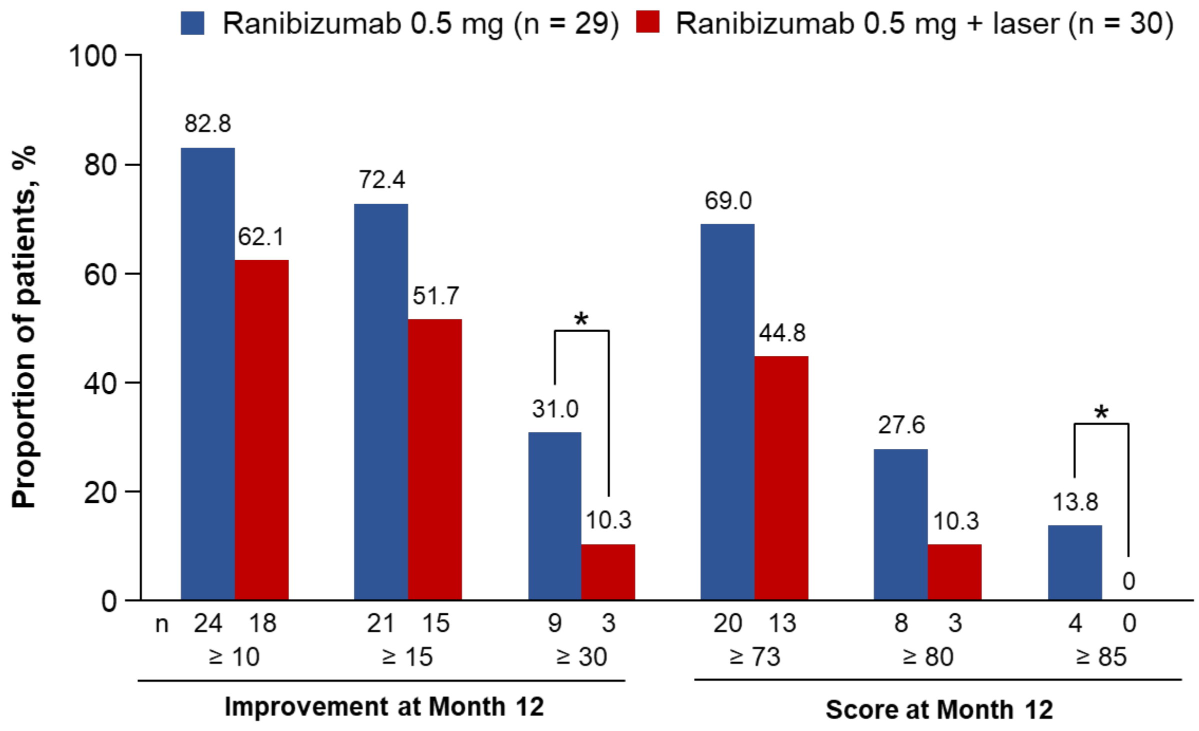

Supplementary Fig. S1. BCVA in the study eye (full analysis set, LOCF): Patients with improvement in the study eye vs baseline at Month 12.

BCVA best corrected visual acuity, *LOCF* last observation carried forward.

*$p < 0.05$

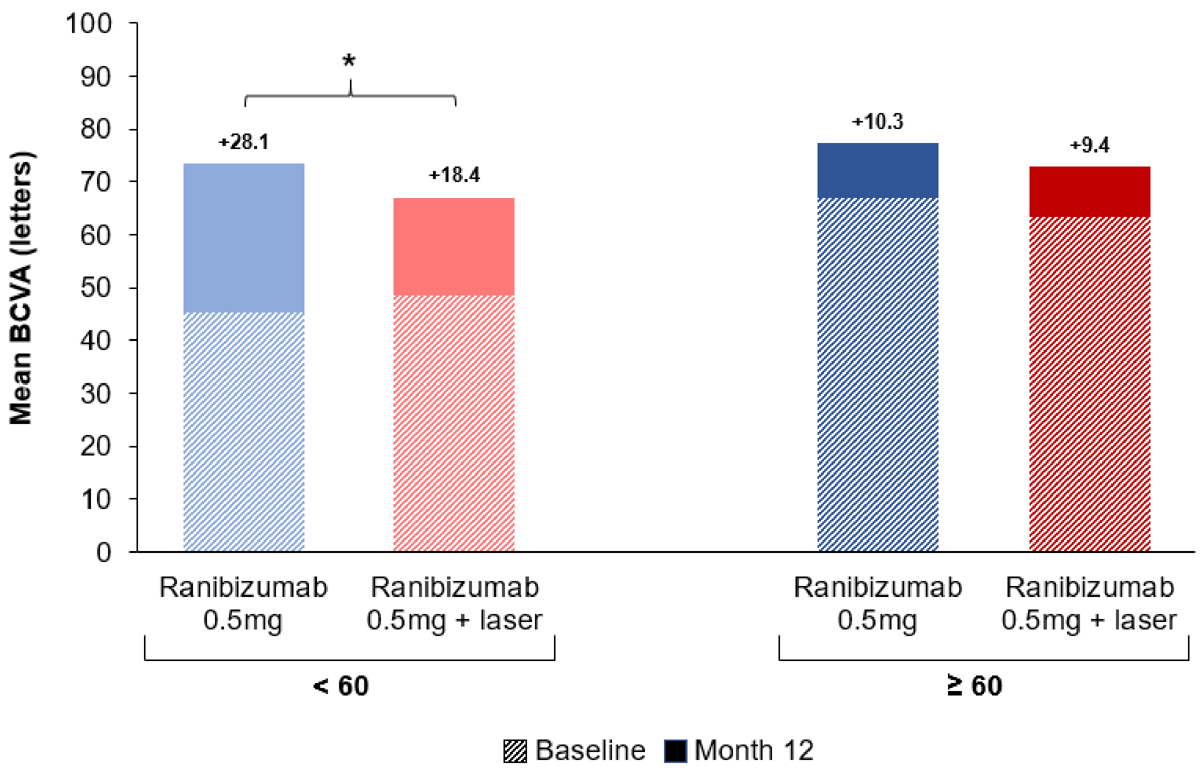

Supplementary Fig. S2. BCVA in the study eye (full analysis set, LOCF): Mean change from baseline in BCVA (letters) at Month 12 in patients with baseline BCVA < 60 and ≥ 60 letters.

BCVA best corrected visual acuity, *LOCF* last observation carried forward.

*$p < 0.05$

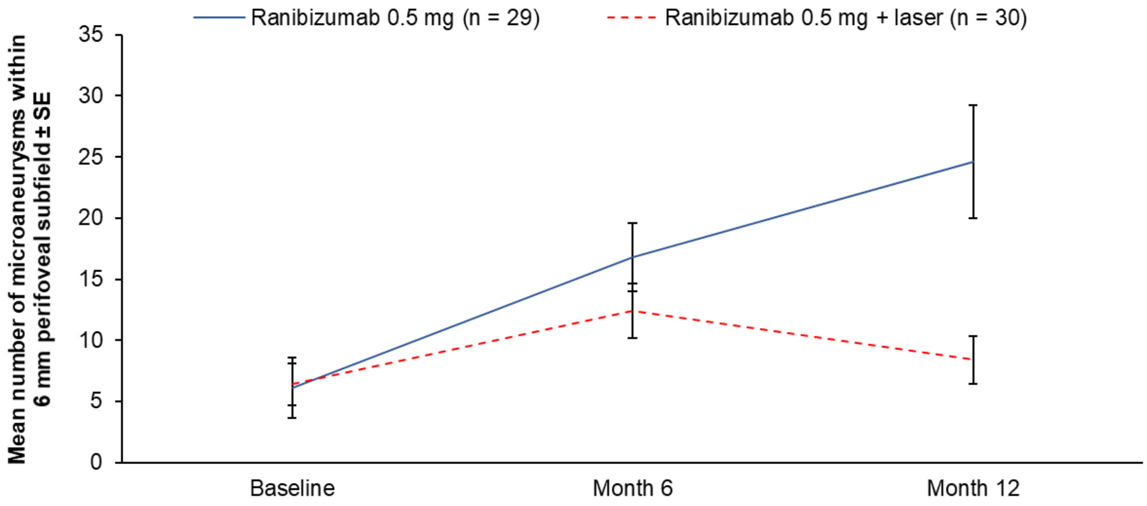

Supplementary Fig. S3. Exploratory analysis of changes in foveal and macular leaking microaneurysms, 6 mm subfield.

SE standard error.

Supplementary Table S1. Summary of best corrected visual acuity in the study eye, absolute values and change from baseline at Month 12 (full analysis set, LOCF)

**Variable**	**Ranibizumab 0.5 mg**<br>**n = 29**	**Ranibizumab 0.5 mg**<br>**+ laser**<br>**n = 30**
BCVA (logMAR)		
Baseline	29	30
Mean (SD)	0.6 (0.2)	0.6 (0.2)
Month 12, n	29	29
Mean (SD)	0.1 (0.2)	0.1 (0.2)
Change from baseline at Month 12, n	29	29
Mean (SD)	−0.5 (0.3)	−0.4 (0.2)
Difference in means (SE)	0.1 (0.1)	
95% CI	−0.1, 0.2	
ANOVA estimate (combination vs monotherapy)		
LS mean (SE)	0.5 (0.0)	−0.4 (0.0)
Difference in the means (SE)	0.1 (0.1)	
p value[a]	0.271	

ANOVA analysis of variance, *BCVA* best corrected visual acuity, *CI* confidence interval, *IQR* interquartile range, *LOCF* last observation carried forward, *logMAR* log of the minimal angle of resolution, *LS* least squares, *SD* standard deviation, *SE* standard error of the mean.

[a]ANOVA t-test (stratified) for treatment difference vs monotherapy.

Supplementary Table S2. Summary of retinal sensitivity, absolute values and change from baseline at Month 12 (full analysis set, observed)

**Variable**	**Ranibizumab 0.5 mg**<br>**n = 29**	**Ranibizumab 0.5 mg**<br>**+ laser**<br>**n = 30**
Total area		
Baseline, n	19	16
Mean (SD)	16.2 (5.3)	15.7 (3.3)
Month 12, n	26	18
Mean (SD)	21.7 (4.6)	18.9 (3.2)
Change from baseline at Month 12, n	19	14
Mean (SD)	4.9 (3.1)	3.0 (2.7)
Foveal area		
Baseline, n	19	16
Mean (SD)	12.9 (6.0)	11.5 (5.6)
Month 12, n	26	19
Mean (SD)	21.0 (5.1)	22.2 (14.9)
Change from baseline at Month 12, n	19	15
Mean (SD)	7.3 (4.1)	10.9 (14.4)
Affected side		
Baseline, n	19	16
Mean (SD)	11.4 (7.4)	10.5 (4.9)
Month 12, n	26	18
Mean (SD)	17.6 (6.9)	14.5 (5.9)
Change from baseline at Month 12, n	19	14
Mean (SD)	5.5 (5.2)	3.6 (5.1)
Unaffected side		
Baseline, n	19	16
Mean (SD)	20.6 (5.6)	20.9 (3.8)
Month 12, n	26	18
Mean (SD)	25.8 (2.8)	23.8 (4.2)
Change from baseline at Month 12, n	19	14
Mean (SD)	5.1 (5.6)	2.9 (3.8)

SD standard deviation.
